# Recent Advances in Biosorption of Copper and Cobalt by Filamentous Fungi

**DOI:** 10.3389/fmicb.2020.582016

**Published:** 2020-12-21

**Authors:** Leonce Dusengemungu, George Kasali, Cousins Gwanama, Kennedy Ochieng Ouma

**Affiliations:** ^1^School of Mathematics and Natural Sciences, The Copperbelt University, Kitwe, Zambia; ^2^School of Natural Resources, The Copperbelt University, Kitwe, Zambia

**Keywords:** copper, cobalt, filamentous fungi, biosorption, bioremediation

## Abstract

Copper (Cu) and Cobalt (Co) are among the most toxic heavy metals from mining and other industrial activities. Both are known to pose serious environmental concerns, particularly to water resources, if not properly treated. In recent years several filamentous fungal strains have been isolated, identified and assessed for their heavy metal biosorption capacity for potential application in bioremediation of Cu and Co wastes. Despite the growing interest in heavy metal removal by filamentous fungi, their exploitation faces numerous challenges such as finding suitable candidates for biosorption. Based on current findings, various strains of filamentous fungi have high metal uptake capacity, particularly for Cu and Co. Several works indicate that *Trichoderma, Penicillium*, and *Aspergillus* species have higher Cu and Co biosorption capacity compared to other fungal species such as *Geotrichum, Monilia*, and *Fusarium*. It is believed that far more fungal species with even higher biosorption capability are yet to be isolated. Furthermore, the application of filamentous fungi for bioremediation is considered environmentally friendly, highly effective, reliable, and affordable, due to their low technology pre-requisites. In this review, we highlight the capacity of various identified filamentous fungal isolates for biosorption of copper and cobalt from various environments, as well as their future prospects.

## Introduction

The biosorption of copper and cobalt by filamentous fungi has received a great deal of attention in recent years, as an emerging technology for minimizing the distribution of these heavy metals from mining wastes, e-wastes, and industrial wastewaters (Hussein et al., 2004; Dhankhar and Hooda, 2011; Ahemad and Kibret, 2013). The presence and persistence of elevated levels of heavy metals in the environment causes harmful effects on humans and other organisms mainly due to moderate accumulation over time. Leenu and Sheela (2016) reported that copper and cobalt bioaccumulation in *Eichhornia crassipes* (Mart) affects the plant growth, especially physiological processes and metabolism. The results of their study indicated that 800 μM of Cu and Co treatment had detrimental effects on the growth rates of *E. crassipes* especially on the development of leaves, stomata, and petioles. The reduced growth and size of aquatic fauna due to the presence of significantly harmful levels of heavy metals has also been observed by Nakayama et al., 2010).

**FIGURE 1:**
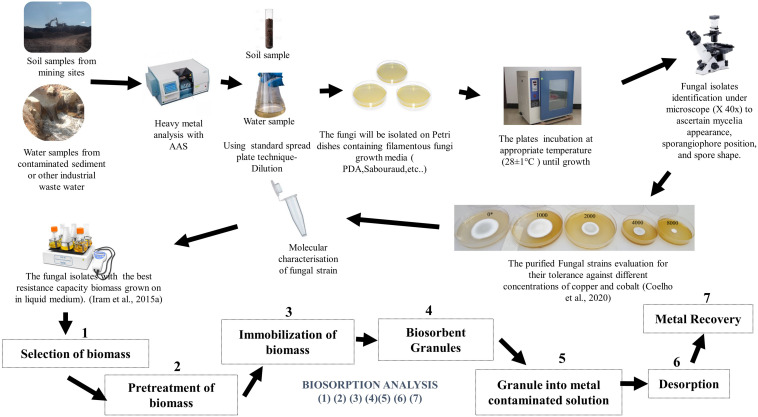
Typical laboratory schematic process of heavy metal biosorption by filamentous fungi (Biosorption analysis is accomplished from experiment 1 to 7).

Numerous reports have demonstrated that in heavy metal polluted environments, microbial populations develop the ability to adapt to the high contamination levels (Forster, 2003; Narendrula-Kotha and Nkongolo, 2017; Coelho et al., 2020). Nevertheless, there are concerns over heavy metal pollution from industries including mining, electroplating, dyeing, and tannery (Shukla and Vankar, 2014; Kapahi and Sachdeva, 2019). To address the effect of heavy metal contamination, chemical methods such as ion-exchange, electrochemical treatment, and chemical precipitation have been developed to control excess heavy metals in wastewater and agricultural fields (Shukla and Vankar, 2014). These conventional methods are usually expensive and ineffective whenever heavy metal concentrations in the environment occur in very low concentrations. Therefore, the option to complement and integrate conventional methods with biological methods such as filamentous biosorption and bioaccumulation is becoming increasingly attractive (Kapahi and Sachdeva, 2019). The basic and simplified laboratory process for fungal biosorption is demonstrated in Figure 1.

Filamentous fungi are ubiquitous in the environment. The diameter of fungal hyphae is between 2 and 10 μm while a fungal mycelium comprises an interconnected network of mm- to cm-long hypha. Due to their vegetative features, filamentous fungi are among the most economical biofriendly biosorbents available today (Shah et al., 2020). Also, fungal biomasses can be easily produced by using cheap growth media. Moreover, they are easy to grow at a massive scale, with a considerably high biomass yield. Fungi are highly abundant in industrial waste products after fermentation and possess numerous advantages in comparison to other microorganisms such as bacteria (Table 3; Dhankhar and Hooda, 2011; Cárdenas González et al., 2019). According to Cerino-córdova et al. (2012), *Aspergillus terreus* Thom (1918), has the capacity to biosorb, 68.52% of copper from aqueous solutions. Although these results indicate that there is a possibility of using *Aspergillus* terreus as a biosorbent, its capacity is not yet satisfactory and more research are needed to enhance its chelating ability. Luckily, the investigation of biosorption of Cu and Co by *Aspergillus* species and other filamentous fungi can be accomplished at a minimal cost (Drahansky et al., 2016).

The distinctive characteristics of the rigid cell wall of fungi (Table 1) composed of mineral ions and nitrogens, as well as its filamentous branching growth habit, are what make fungi such great decomposers and great candidates for the biosorption of heavy metals (Naveena and Latha, 2018). Filamentous fungi typically accumulate metal ions into their mycelium and spores through the mechanisms that involve the fungal cell wall (intracellular/biosorption and extracellular/adsorption processes) which also plays a significant role for the existence and performance of the fungi, as well as energetic uptake and valence conversion (Siddiquee et al., 2015; Igiri et al., 2018; Naveena and Latha, 2018). The rigid cell wall has numerous advantageous characteristics. For instance, the chitin contains chitosan, a polymer of N-acetyl D-glucosamine, which enhances metal uptake and binding properties (Table 1; Naveena and Latha, 2018).

**TABLE 1:** Types of Microorganisms used in bioremediation and their cell wall characteristics.

Microorganisms	Cell wall	References
	The common groups in the cell wall are: amine, hydroxide, carboxyl, sulfate, and phosphate, sulfhydryl	Gupta et al., 2000
Fungi	Has chitin, mineral ions, lipids which contain polysaccharides and glycoproteins, which also has: amine, imidazole, phosphate (PO4^3–^), sulfate, sulfhydryl, and hydroxyl (COO^–^) groups (High percentage of cell wall material compared with other microorganisms)	Lakshmi et al., 2018
Algae	Algal cell walls are mainly cellulosic: the cell walls of brown algae contain fucoidin and alginic acid. The alginic acid offers anionic carboxylate and sulfate sites at neutral pH	Gupta et al., 2000
Bacteria	An outer layer of lipopolysaccharide (LPS), phospholipids and proteins and Functional groups: ketones, aldehydes, and carboxyl groups	Lakshmi et al., 2018
Cyanobacteria	Peptidoglycans which consist of linear chains of the disaccharide N-acetylglucosamine-β1,4-N-acetylmuramic acid with peptide chains	Vannela and Verma, 2006
Yeast	Contain a microfibrillar structure which is composed of more than 90% polysaccharides.	Lakshmi et al., 2018

In addition to their ability to bind metals and withstand high variations in nutrient concentrations and other environmental parameters such as pH, anoxic, and temperature, filamentous fungi are usually used in fermentation industries (Dhankhar and Hooda, 2011; Lakshmi et al., 2018). Thus, it is practical to produce and use fungal biomass at large scale for industrial purposes such as bio-removal of metal ions from highly contaminated effluents. According to Lakshmi et al. (2018), the high number of fungal biosorbents is non-toxic and therefore, they are generally safe and easy to handle.

Microbial diversity is highly influenced by the level of contamination as well as contamination events in different locations. It has been demonstrated that long-term contamination is a crucial factor that determines the quality of water, soil, and microbial community besides environmental circumstances and soil characteristics (Crognale et al., 2017). Some studies have confirmed that prolonged contamination of a particular area positively impacts the abundance and immunity of microbial communities (Brandl et al., 1999; Sutton et al., 2013). In contrast, short-term pollution has a deleterious effect on microbial diversity and density (Bourceret et al., 2016). On the other hand, controversial studies have reported that both short-term and long-term exposure of soil to heavy metals results in a detrimental effect on soil microbial diversity (e.g., Narendrula-Kotha and Nkongolo, 2017). However, the fungal community dominated by *Ascomycota* isolated from aged multi-polluted soils was confirmed to adapt to rich microbial diversity compared to non-polluted areas (Bourceret et al., 2016; Crognale et al., 2017). In this regard, in-depth investigation of fungal tolerance and diversity in heavily contaminated environments especially mining sites is of fundamental importance since fungi represent a large proportion of biodiversity and biological matter. These saprophytes also play an essential role in maintaining proper functioning of the ecological system (Crognale et al., 2017). Taking into account that fungi and other microbial communities are crucial in biogeochemical recycling and influence the availability of metals, it is necessary to acquire in-depth knowledge of the taxonomic and functional diversity of fungal communities in heavy metal contaminated sites (Bourceret et al., 2016; Crognale et al., 2017; Narendrula-Kotha and Nkongolo, 2017).

Several filamentous fungi have been associated with the removal of copper and cobalt from aqueous environments (Table 2). Fungal biomass in large quantities can facilitate clean-up of contaminated waters and restoration of contaminated ecosystems. According to studies by Wang and Chen (2009) and Fu and Wang (2011), fungal biomass has a high affinity for copper and cobalt uptake. Therefore, they are relatively suitable for use in the decontamination of polluted sites. Propositions have been made to use filamentous fungi in combination with other microorganism matrices, such as bacteria, to increase biosorption efficiency. It is suggested that how the combination of fungi and other microorganisms (bacteria, algae, yeast) harvest their full potential for heavy metal biosorption remains a novel research area. The resistance mechanisms developed by these microorganisms mainly for their survival shows that they are highly efficient for detoxifying metal ions in aqueous solutions (Xie et al., 2010; Mohammadian et al., 2017). Developing new technologies for bioremediation is especially relevant in areas with heavy mining activities such as Zambia and The Democratic Republic of the Congo, where copper and cobalt pollutions pose serious public health and environmental hazard. Thus, the restoration of contaminated terrestrial and aquatic environments is necessary in order to reduce the severe impact of copper and cobalt pollution in these areas (Kapungwe, 2013; Ilunga wa Ilunga et al., 2015; Chileshe et al., 2019).

**TABLE 2:** The application of different filamentous fungi for the removal of Cu and Co from different environmental matrices.

Heavy metal	Fungal strain	Environmental matrix	Country	Removal capacity (mg/g)	References
Cu	*Acremonium persicinum* (Nicot) W. Gams, (1971)	Soil samples collected from the surface layer (0–30 cm) of the 69 Anguran lead-zinc mining	Iran	50–100	Mohammadian et al., 2017
	*Alternaria alternata* Fr. Keissl. (1912)	Samples of water and sediment from five contaminated sites in the Moghogha river (Tangier)	Morocco	–	Ezzouhri et al., 2009
	*Alternaria chlamydosporigena* Woudenb. Crous (2013)	Soil samples collected from the surface layer (0–30 cm) of the 69 Anguran lead-zinc mining	Iran	50–100	Mohammadian et al., 2017
	*Alternaria tenuis* Nees (1816)	–	United Kingdom	22.4	Somers, 1963
	*Aspergillus awamori* (Nakaz, 1907)	The Culture Collection of the Institute of Microbiology	Bulgaria	–	Tsekova, 2000
	*Aspergillus carbonarius* (Bainier) Thom (1916)	Obtained from a fermentation process	Canada	11.6	Al-Asheh and Duvnjak, 1995
	*Aspergillus flavus* Link (1809)	Soil samples	Pakistan	93.65	Iram et al., 2015
	*Aspergillus flavus* ED4	Wise park industrial area, Palakkad	India	10	Jayaraman and Arumugam, 2014
	*Aspergillus fumigatus* Fresen. (1863)	–	China	40	Yin et al., 2011
	*Aspergillus niger* Tiegh. (1867)	An open water system at the Langat River, Selangor	Malaysia	20.910 + 0.581	Iskandar et al., 2011
	*Aspergillus oryzae* (Ahlb.) Cohn (1884)	Carolina Biological Supply Company	United States	13.8	Huang et al., 1991
	Aspergillus tamarii NRC3 Kita (1913)	Egyptian soil	Egypt	–	Saad et al., 2019
	Aspergillus terreus Thom (1918)	–	India	160–180	Gulati et al., 2002
		–	Mexico	15.24	Cerino-córdova et al., 2012
	*Aspergillus ustus* (Bainier) Thom and Church (1926)	Soil samples	Saudi Arabia	0.6494 ± 0.006	Alothman et al., 2019
	*Aspergillus versicolor* (Vuill.) Tirab. (1908)	Soil samples	Turkey	10.08 ± 0.7	Taştan et al., 2010
	*Fomitopsis meliae* (Underw.) Gilb. (1981)	Gemstone and gold mining sites in Southwestern, Nigeria	Nigeria	0.78	Oladipo et al., 2018
	*Fusarium flocciferum* Corda (1828)	–	Portugal	2.3	Raftos and Radford, 2002
	*Fusarium oxysporum* Schltdl. (1824)	–	Romania	2.26	Simonescu and Ferdeş, 2012
	*Fusarium solani* Mart.) Appel and Wollenw. (1910)	Topsoil samples near petrol pumps	United Kingdom	0.66 ± 0.20	Hong et al., 2010
	*Fusarium verticillioides* (Sacc.) Nirenberg (1976)	Soil samples collected from the surface layer (0–30 cm) of the 69 Anguran lead-zinc mining	Iran	100–150	Mohammadian et al., 2017
	*Geotrichum* sp. Link (1809)	Agricultural field soil treated with municipal wastewater/industrial effluent	India	0.7	Zafar et al., 2007
	*Hypocrea lixii* Pat. (1891)	Topsoil samples near petrol pumps	United Kingdom	0.54 ± 0.09	Hong et al., 2010
	*Monilia* sp. Bonord. (1851)	Agricultural field soil treated with municipal wastewater/industrial effluent	India	0.6	Zafar et al., 2007
	*Mucor rouxii* (Calmette) Wehmer (1900)	From the company Citric Belge (Tirlemont, Belgium).	South Korea	200	Baik et al., 2002
	*Penicillium brevicompactum* Dierckx (1901)	The Collection of the Institute of Microbiology at the Bulgarian Academy of Sciences.	Bulgaria	25.32	Tsekova et al., 2007
	*Penicillium* Chrysogenum XJ-1 Thom (1910)	Laboratory strain (GU733711)	China	42.83 ± 0.57	Xu et al., 2018
	*Penicillium cyclopium* Westling (1911)	The Collection of the Institute of Microbiology at the Bulgarian Academy of Sciences	Bulgaria	50.0	Ianis et al., 2006
	*Penicillium griseofulvum* Dierckx (1901) (Immobilized)	Supplied by Cadila Healthcare Pvt, Ltd., Gujarat	India	20.47	Shah et al., 1999
	*Penicillium griseofulvum* Dierckx (1901) (free)	Supplied by Cadila Healthcare Pvt, Ltd., Gujarat	India	1.51	Shah et al., 1999
	*Penicillium italicum* Wehmer (1894)	Laboratory strain	United Kingdom	–	de Rome and Gadd, 1987
	*Penicillium notatum* Westling (1911)	99.999% pure foils obtained from Alfa Products,	United States	80	Siegel et al., 1983
	*Penicillium ochrochloron* Biourge (1923)	–	Japan		Fuwa et al., 1977
	*Penicillium simplicissimum* (Oudem.) Thom (1930)	An open water system at the Langat River, Selangor	Malaysia	10.767 ± 0.416	Iskandar et al., 2011
	Penicillium spinulosum Thom (1910)	–	–	0.4–2	Townsley and Ross, 1985
	*Rhizopus arrhizus* A. Fisch. (1892)	From The United States Department of Agriculture Culture Collection	Turkey	7.32	Aksu, 2003
	*Rhizopus arrhizus* A. Fisch. (1892)	The institute of microbial technology, Chandigarh	India	–	Preetha and Viruthagiri, 2007
	*Rhizopus microsporus* Tiegh. (1875)	Gemstone and gold mining sites in Southwestern, Nigeria	Nigeria	1.02	Oladipo et al., 2018
	*Rhizopus oryzae* Went and Prins.Geerl. (1895)	From the company Citric Belge (Tirlemont, Belgium).	South Korea	101	Baik et al., 2002
	*Seimatosporium pistaciae* Crous and Mirab. (2014)	Soil samples	Iran	50–100	Mohammadian et al., 2017
	*Talaromyces helicus* (Raper and Fennell) C.r. Benj. (1955)	Co-contaminated sludge of the East Channel, near the YPF-oil Refinery, La Plata, Argentina.	Argentina	–	Romero et al., 2006
	*Trichoderma asperellum* Samuels, Lieckf. and Nirenberg (1999)	An open water system	Malaysia	15.374 ± 0.370	Iskandar et al., 2011
	*Trichoderma atroviride* P. Karst. (1892)	Sewage sludge (Water treatment plant)	Spain	–	López Errasquín and Vázquez, 2003
	*Trichoderma ghanense* Yoshim.Doi, Y. Abe and Sugiy. (1987)	Gold and gemstone mine site soils	Nigeria	1.27	Oladipo et al., 2018
	*Trichoderma harzianum* Rifai (1969)	Contaminated mining soils	–	650	Mohammadian et al., 2017
	Trichoderma SP2F1	Sediment form Phenchala River	Malaysia	28.75	Ting and Choong, 2009
	*Trichoderma virens* (J.H. Mill., Giddens and A.A. Foster) Arx (1987) (PDR-28)	Rhizosphere soil	South Korea	–	Babu et al., 2014
	*Trichoderma viride* Schumach. (1803)	The soil collected from a metal polluted site in New Delhi	India	3.4	Anand et al., 2006
Co	*Aspergillus flavus* Link (1809)	Obtained from the mycology and plant pathology section, Department of Botany, Osmania University, Hyd. N. crassa wild type (4200 a)	India	50.8	Rashmi et al., 2004
	*Aspergillus niger* Tiegh. (1867)	The polluted air in a fuel station, near to the Faculty of Chemical Science, belonging to the Autonomous University of San Luis Potosı (San Luis Potosi, Mexico)	Mexico	33	Cárdenas González et al., 2019
	*Aspergillus tamarii* Kita (1913) NRC3	Egyptian soil	Egypt	–	Saad et al., 2019
	*Fusarium oxysporum* Schltdl. (1824)	Fungal Genetics Stock Centre (Kansas City, KS	India	–	Akthar et al., 1996
	*Geotrichum* sp. Link (1809)	Agricultural field soil treated with municipal wastewa-ter/industrial effluent	India	0.1	Zafar et al., 2007
	*Monilia* sp. Bonord. (1851)	Agricultural field soil treated with municipal wastewa-ter/industrial effluent	India	0.1	Zafar et al., 2007
	Mortierella Coem. (1863) SPS 403	From serpentine soil samples	India	1036.5 + 39.6	Pal et al., 2006
	Mucor recemosus Fresen. (1850)	Obtained from the mycology and plant pathology section, Department of Botany, Osmania University, Hyd. N. crassa wild type (4200 a)	India	64	Rashmi et al., 2004
	Neurospora Shear and B.O. Dodge (1927)	Fungal Genetics Stock Centre (Kansas City, KS	India	–	Akthar et al., 1996
	Neurospora crassa CSM-1 and CSM-II Shear and B.O. Dodge (1927)	Obtained from the mycology and plant pathology section, Department of Botany, Osmania University, Hyd. N. crassa wild type (4200 a)	India	44–29	Rashmi et al., 2004
	*Paecilomyces catenlannulatus*	Culture collection of microbiology laboratory, University of Huangshan, China	China	–	Li et al., 2014
	*Paecilomyces* sp. Bainier (1907)	The polluted air in a fuel station, near to the Faculty of Chemical Science	Mexico	24	Cárdenas González et al., 2019
	Penicillium brevicompactum Dierckx (1901)	The Collection of the Institute of Microbiology at the Bulgarian Academy of Sciences.	Bulgaria	54.64	Tsekova et al., 2007
	*Penicillium citrinum* Thom (1910)	Obtained from the mycology and plant pathology section, Department of Botany, Osmania University, Hyd. N. crassa wild type (4200 a)	India	126.4	Rashmi et al., 2004
	*Penicillium* sp. Link (1809)	The polluted air in a fuel station, near to the Faculty of Chemical Science	Mexico	37	Cárdenas González et al., 2019
	*Penicillium* sp. AND 104	From serpentine soil samples of Saddle hills, Chidyatapu and Rutland of Andaman Islands, India.	India	882.4 + 16.4	Pal et al., 2006
	*Pythium* sp.CTS 703 (Pringsh.1858)	From serpentine soil samples	India	670.6 + 10.7	Pal et al., 2006
	*Rhizopus arrhizus* A. Fisch. (1892)	PFBl-Laboratory stock isolates	India	190	Suhasini et al., 1999
	*Rhizopus chinensis* Saito (1904)	Obtained from the mycology and plant pathology section, Department of Botany, Osmania University, Hyd. N. crassa wild type (4200 a)	India	11.5	Rashmi et al., 2004
	Talaromyces helicus (Raper and Fennell) C.r. Benj. (1955)	Co-contaminated sludge of the East Channel, near the YPF-oil Refinery, La Plata, Argentina.	Argentina	–	Romero et al., 2006
	*Trichoderma* sp. SPS 404 Pers. (1801)	From serpentine soil samples	India	660.4 + 15.0	Pal et al., 2006
	*Trichoderma viride* Schumach. (1803)		India	83.2	Rashmi et al., 2004

**TABLE 3:** The primary advantages and disadvantages of using filamentous fungi in bioaccumulation of heavy metals.

Characteristics	Advantages	Disadvantages	References
Temperature	Fungi can grow on various carbon sources such as fatty acids and oils which also indicate the capacity to withstand high temperature up to 40°C	High efficiency is only observed at high temperatures, Inadequate for cold climates	Ban et al., 2001; Iskandar et al., 2011
pH	They can grow within a wide range of pH (2–9) which is an advantage since most wastewater varies in pH concentration	Some time adjusting pH can increase the efficiency level	Ozsoy, 2010
Rate of removal	Fast and efficient compared to other means of bioremediation/maximum efficiency for removal of low concentrations of heavy metals from wastewater	Depends on the sources of biomass as well as the concentration of heavy metals in wastewaters	Barakat, 2011
Selectivity	Depending on the type of heavy metal, can be modified through bioengineering	Low selectivity	Lakshmi et al., 2018; Igiri et al., 2018
Energy requirement	Low	Requires oxygen	Harms et al., 2011; Lakshmi et al., 2018
Effectiveness	Highly effective (They can supply a high range of organic substrates		Fu and Wang, 2011
Cost	Low, can be produced at the minimum operating cost	–	Fan et al., 2008
Reuse and Regeneration	It protects the environments allowing recovery of metals and reuses	–	Siddiquee et al., 2015
Long-range transport	Fungi adapt with resource heterogeneity by translocating resources between different parts of their mycelium	–	Harms et al., 2011
Contamination	-Low sludge generation compared to other removal techniques such as precipitation	Might produce toxic substances at low rates compared with other conventional methods	Fu and Wang, 2011
Variability	Highly variable (can be used in an extremely contaminated environment, too acidic, or too dry) and produce a relatively large amount of biomass	–	Fu and Wang, 2011; Harms et al., 2011

The present review attempts to enumerate the filamentous fungal species/strains/groups with comparatively high capacity to absorb copper and cobalt metal ions from contaminated environments. Progress in the development of technology to reduce the effect caused by copper and cobalt pollution through filamentous fungi biosorption is also discussed. A wide variety of filamentous fungi described in this article can be easily employed to create advanced heavy metal removal systems together with other biotreatment methods for maximum metal uptake efficiency in the future.

## Potential Filamentous Fungi Species and Their Properties in Biosorption of Copper and Cobalt

Biosorption refers to the natural capacity of biological materials to accumulate heavy metals from diverse locations (mineral ores, mining wastes, industrial wastes and wastewaters, etc.). Biosorption occurs naturally in the environment, and recently, researchers have experimented with the ability of microorganisms to biosorb heavy metals and possibly valuable metals from several locations (Iram et al., 2015). Biosorption is one aspect of biohydrometallurgy based on the interactions between living microorganisms and metallic ions (Iskandar et al., 2011). Two principal bacteria families are used in biohydrometallurgy. These are the chemolithotrophic bacteria such as *Thiobacillus thiooxidans* Waksman and Joffe (1922) and *Thiobacillus caldus* Hallberg and Lindström (1995). Other heterotrophic microorganisms that can also be used include fungi, yeast, and other associated species (Johnson, 2014).

For reasons not well understood, the role of fungi species in the biosorption of heavy metals and other biohydrometallurgy purposes has been less regarded for extensive scale application (Borja et al., 2016; Liang and Gadd, 2017). This is despite their undeniable advantages over their counterparts, the acidophilic bacteria, such as their ability to grow over a wide range of pH from 1.0 to 9, and their higher sensitivity and capacity to accumulate more heavy metals (0.40–700 mg/g) compared to bacteria biosorption (0.70–500 mg/g) (Ahemad and Kibret, 2013). Numerous researches have published findings on the biosorption of heavy metals by filamentous fungi but mostly under controlled laboratory-scale environments (Zafar et al., 2007; Ezzouhri et al., 2009; Siddiquee et al., 2013; Mohammadian et al., 2017). To understand, clearly and in detail, the progress in biosorption of heavy metals by filamentous fungi, our review has focused mainly on the biosorption of copper and cobalt from heavy metal contaminated environments (Table 2).

### *Aspergillus* Species

The *Aspergillus* species are filamentous fungi ubiquitous in the environment. They possess many industrial applications, including practical applications in biosorption of heavy metals from contaminated sites. These species have enormous ability to create a metal sink coupled with their capacity to produce organic acids that can bioleach metals. There are four popular species of *Aspergillus*: *A. niger, A. flavus, A. versicolor*, and *A. tamarii* NRC 3 used for biosorption (Oladipo et al., 2018; Table 2).

#### *Aspergillus flavus* Link (1809)

*Aspergillus flavus* isolated from effluent samples obtained from wise park industrial area, India, was examined for its biosorption ability to uptake Cu (II). The results demonstrated that 80% of copper could be biosorbed from an aqueous solution (Jayaraman and Arumugam, 2014). The findings confirmed that *A. flavus* can be classified among potential biosorbents for the clean-up of copper contaminated environments. Their results showed that biosorbents after the removal of copper or other heavy metals could be reused or regenerated again. In addition, the above results agree with the findings by Rao et al. (2002), which indicated that *A. flavus* (DSF-8) could also be used in the bioleaching of copper.

In another study by Qayyum et al. (2019), *A. flavus* isolated from contaminated soil has shown high effectiveness in the biosorption of heavy metals. The study concluded that with sucrose as a carbon source at 30°C and pH of 7, bioleaching of Pb, Cd, and Zn with *A. flavus* was possible. It was also suggested that the use of *A. flavus* for contaminated soil bioremediation could be applied for maximum results whenever the optimal conditions for the growth of *A. flavus* are provided.

Synthetic flocculants can be employed in wastewater treatment, but they mainly have a disadvantage due to their toxicity (David et al., 2019). Therefore, in an attempt to minimize the toxicity and use a more ecofriendly and biodegradable flocculant, *A. flavus* and *Paenibacillus elgii* Kim (2004) isolated from wastewater were found more suitable in the production of microbial flocculants which can be used widely for the downstream treatment of wastewater (David et al., 2019).

#### *Aspergillus versicolor* (Vuill.) Tirab. (1908)

*Aspergillus versicolor* has demonstrated significant affinity to heavy metals under certain pH ranges. For instance, it has been demonstrated that for *A. versicolor*, maximum pH values for optimum heavy metal bioaccumulation are around 6 for 50 mg/L of Cr (VI) and Ni (II) while pH 5 is suitable for Cu (II) ions with the 99.89, 30.05, and 29.06% removal efficiencies, respectively (Taştan et al., 2010). Although the biosorption capacity of *A. versicolor* is still unsatisfactory for Cu removal from aqueous solution, it has a promising future application in biosorption of heavy metals.

#### *Aspergillus niger* Tiegh. (1867)

Previous studies have tested *Aspergillus niger* for its removal capacity of heavy metals from industrial as well as swine wastewaters. The results indicated that *A. niger* could absorb 45.54% of Pb and 59.67% of Cd and 91% of the copper from the swine wastewaters (Pal and Basumajumdar, 2002). A study by Kapoor et al. (1999) which also evaluated the ability of live *A. niger* biomass to uptake Pb, Cd, Cu and Ni from wastewaters and found that the removal of these heavy metals was higher when the *Aspergillus* biomass was used compared to granular activated carbon (E-400). The above experiments demonstrate that *A. niger* can be used to accumulate metals from the contaminated environments. However, more research is needed to increase the biosorption capacity of *A. niger* and enhance more efficient use of the fungal.

#### *Aspergillus tamarii* NRC 3 Kita (1913)

*Aspergillus tamarii* NRC 3 obtained from contaminated Egyptian soil was used as a biosorbent for the bioremoval and bio-recovery of heavy metals (Saad et al., 2019). From the findings, live *A. tamarii* NRC 3 biomass could absorb 90.94% Cu (II), 29.13 of Pb (II), 60% of Co (II), 40% of Ni (II), 34.47% of Fe (II), and 11.45% of Cr (II) respectively. Their findings concluded that *A. tamarii* NRC 3 biomass, even though its biosorption process is affected by temperature, pH, metal ions concentration, and changing time through the optimization of these parameters, the performance of *A. tamarii* can be improved (Saad et al., 2019).

### *Trichoderma* Species

The *Trichoderma* spp. are among the highly diverse community of filamentous fungi worldwide. They contain teleomorphs and have been classified into the order of Hypocreales in the Ascomycete division. These species are also among the most culturable fungi whose group members are considered very popular for their biocontrol capacity, which can facilitate to eliminate a broad range of plant pathogenic fungi (Table 2; Nongmaithem et al., 2016). They are also known for plant growth enhancement (Yedidia et al., 1999), inducing oriented and systematic plant defense responses against pathogens (Harman et al., 2004; Nongmaithem et al., 2016). Since the *Trichoderma* spp. are extremely interactive in soil and plant roots, they play a significant role in the environment by facilitating in the decomposition of plant residues, biodegradation of industrial chemicals as well as bioremoval/bioaccumulation of a wide range of heavy metals from wastewaters and soil, due to their unique cell-wall characteristics which are mainly composed of glucan polymers and chitin (Nongmaithem et al., 2016; Akhtar and Mannan, 2020).

#### *Trichoderma atroviride* P. Karst. (1892)

*Trichoderma atroviride* has been used for many industrial processes, including the uptake of heavy metals. *Trichoderma atroviride* obtained from sewage sludge was tested for its ability to tolerate high concentrations of Cu, Zn, Cd (López Errasquín and Vázquez, 2003). It was found that the autolyzed mycelia of *T. atroviride* reached the highest values of heavy metal removal while the lowest was recorded when glucose was supplemented. This shows that the composition of the medium has an essential effect on metal uptake (López Errasquín and Vázquez, 2003). In a similar study by Yazdani et al. (2009), *Trichoderma atroviride* extracted from the copper polluted river sediment was found to biosorb between 0.8 and 11.2 mg/g of Cu from potato dextrose liquid medium. From the above results, it is confirmed that *T*. *atroviride* can be used as a bioremediating agent instead of conventional chemical precipitation, chemical oxidation, and reduction methodologies for wastewater treatment.

#### *Trichoderma virens* (J.H. Mill., Giddens and A. A. Foster) Arx (1987)

*Trichoderma virens* is well known for its ability to produce antibiotics and also its mycoparasitic capacity (Harman et al., 2004). Babu et al. (2014) categorized *T. virens* as a metal tolerant fungus because of its potential to tolerate heavy metals such as Pb, Cd, Cu, As, and Zn from the liquid media comprised of 100 mg/L of heavy metals. Their results also suggested that the *T. virens*-PDR-28 can be used for plant biomass production as well as phytostabilisations in mine tailing soils with high heavy metal levels.

#### *Trichoderma ghanense* Yoshim Doi, Y. Abe and Sugiy. (1987)

Oladipo et al. (2018) assessed *Trichoderma ghanense* isolated from a gold and gemstone mine site soil for its tolerance to a high, wide range of heavy metals (As, Fe, Cd, Cu, Pb). Their findings established that *T. ghanense* possesses high resistance to high concentrations of heavy metals, which indicates that it can be used for bioremediation of heavy metal polluted areas. The *T. ghanense* has also been reported to secrete ligninolytic enzymes that can biodegrade heavy metals and antioxidant enzymes with the capacity to minimize oxidative stress due to heavy metal pollution (Akhtar and Mannan, 2020).

#### *Trichoderma* SP2F1

Ting and Choong (2009) isolated *Trichoderma* SP2F1 from the sediment samples from Penchala River, Malaysia, highly contaminated with industrial effluents. Their results have demonstrated that *Trichoderma* SP2F1 was capable of removing Cu (II) in aqueous solutions. The bioaccumulation capacity of viable SP2F1 to remove copper ions was 19.6 mg/g while the non-viable SP2F1 cells removal capacity of 28.75 mg/g. This indicates that both viable and non-viable *Trichoderma* sp. cells can be used to treat sewage wastes or other wastewaters to remove Cu (II). The potential application of the *Trichoderma* spp. for biosorption purposes have been thoroughly reviewed by Gupta et al. (2014) and Sharma et al. (2019).

### *Penicillium* Species

According to Ianis et al. (2006), the young *Penicillium* spp., mycelia, have a far greater capacity for heavy metal biosorption than the old mycelia. The potential of *Penicillium* biomass for removing metal ions from wastewaters was also acknowledged by Zafar et al. (2007) and Iskandar et al., 2011). The data presented from their studies showed that *Penicillium* sp. had higher bioremediation potential for contaminated ecosystems compared to other filamentous fungi strains such as *Aspergillus, Trichoderma, Fusarium, Alternaria, Geotrichum, Rhizopus*, and *Monilia*. Zafar et al. (2007) identified several filamentous fungi, including *Penicillium* spp. from agricultural soil contaminated by heavy metals. Interestingly, it was also noticed that agricultural soil exposed to heavy metal from municipal and industrial wastewaters for more than 20 years possess a wide variety of metal resistant fungi.

#### *Penicillium simplicissimum* (Oudem.) Thom (1930)

*Penicillium* spp. isolated from contaminated sediment in Malaysia were tested for their potential biosorption capacity (Iskandar et al., 2011). The results indicated promising metal ion inhibitory concentrations of Co, Cr, Cu, Cd, and Ni from aqueous solutions. The inhibitory concentrations ranged from 4 to 8 mg/L for *Penicillium simplicissimum* isolates. These results confer with the study by Mohammadian et al. (2017), which also confirmed that *P. simplicissimum* has a higher capacity for Cu (II) uptake compared to other filamentous fungi [*Fusarium verticillioides, Acremonium persicinum, Seimatosporium pistaciae* Crous and Mirab. (2014), *Alternaria chlamydosporigena* Woudenb. and Crous (2013)].

### *Rhizopus arrhizus* A. Fisch. (1892)

The *Rhizopus arrhizus is* zygomycete fungi whose biomass capacity for uptake of metal ions has been previously reported (Tobin et al., 1987; Pal and Basumajumdar, 2002; Cara et al., 2018).

Copper uptake by *R. arrhizus* biomass ranged between 50 and 150 mg/mL (de Rome and Gadd, 1987) while the results by Preetha and Viruthagiri (2007) recorded that under optimized process conditions (4.14 of pH, 37.75°C of temperature, 53.84 mg/L of initial copper ion concentration, and 8.17 g/L of biomass loading) about 98.34% copper removal from aqueous solution can be achieved.

Hence, *Rhizopus arrhizus* is one of the most promising fungi for industrial heavy metal biosorption (Preetha and Viruthagiri, 2007). Research has also established that living microbial biomass of *R. arrhizus* can biosorb 37.785 mg/g of copper with 100 mg biomass and 19.464 mg/g of Cu with 200 mg of biomass (Chauhan et al., 2020).

## Biosorption Mechanisms of Heavy Metals by Microbiota

**FIGURE 2:**
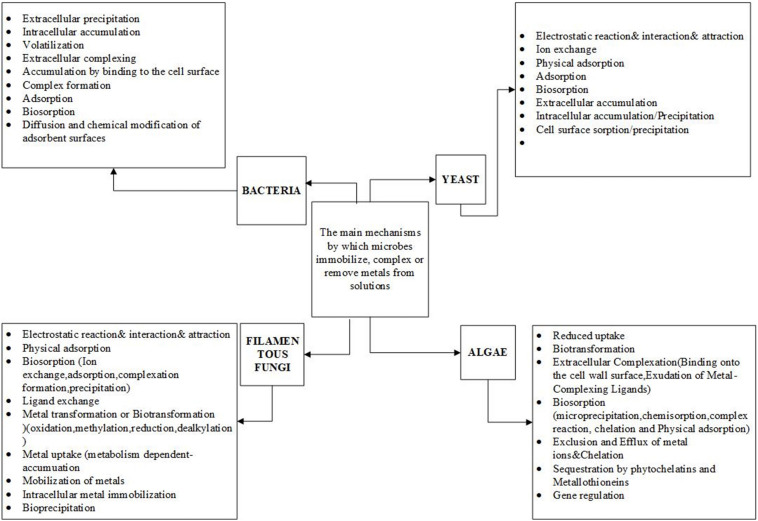
Mechanisms of heavy metals absorption by microorganisms.

The heavy metal accumulation ability of microorganisms such as bacteria (Kumari et al., 2015; Narendrula-Kotha and Nkongolo, 2017), yeast (Bahafid et al., 2017), algae (Mehta and Gaur, 2005; Kipigroch et al., 2016; Bwapwa et al., 2017), and filamentous fungi (Gadd, 1993; More et al., 2010; Liang and Gadd, 2017) has been studied broadly (Figure 2). Their ability for heavy metal removal is relatively higher compared to the conventional non-biological industrial methods. It has been shown that the primary mechanism through which bacteria, filamentous fungi, algae, and yeast accumulate numerous heavy metals is adsorption, which does not require energy for metabolism. Alternatively, in living cells of some of the bacteria, heavy metals are accumulated through absorption, which generally does depend on energy metabolism. Most microbes show the capacity to rapidly absorb a high quantity of heavy metal ions (Ismail and Moustafa, 2016). The primary mechanisms through which microbes immobilize heavy metals are complex and depend on the type of microorganisms (Figure 2).

### Biosorption of Heavy Metals by Fungal Species

**FIGURE 3:**
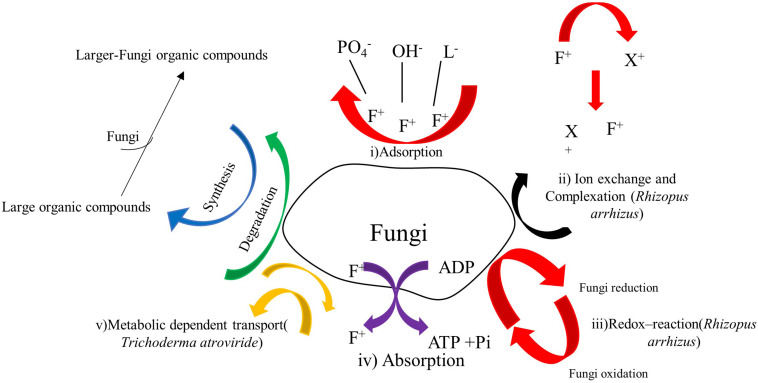
Biosorption mechanisms of copper, cobalt and other heavy metals by filamentous fungi, modified from Ahemad and Kibret (2013) and Siddiquee et al. (2015).

In recent years, the bioremoval mechanisms of heavy metals by some fungal species has been partially studied (Figures 2, 3) and the efficiency of such fungal species to remove heavy metals determined. But still, some mechanisms are not well understood (Suhasini et al., 1999; Taştan et al., 2010; Mathew et al., 2016; Acosta-Rodríguez et al., 2018). The sophisticated cell wall structure profoundly influences the biosorption of heavy metals by fungal species (Table 1). For example, the adsorption mechanism in fungi comprises of metals ions on the cell surface, from where they can be adsorbed into the cell through one mechanism or combined processes (Dhankhar and Hooda, 2011). There is limited research on the fungal mechanisms for biosorption and the exact performance of fungi has not been clarified (Figure 3). It is also noted that little information is known about the effect of combining fungal biomasses with other microbial biomasses. Therefore, it is assumed that the combinations might improve the removal efficiency in some cases (Dhankhar and Hooda, 2011). However, biosorption competence is also determined by filamentous fungi species and adaptability levels depending on the environmental matrix (air, soil, water) where it was obtained (Oladipo et al., 2018).

The mechanism by which filamentous fungi biosorb metal ions out of aqueous solution is complex due to the fungi cell wall structure (Figure 3; Akthar et al., 1996). The biosorption mechanisms can be classified into two categories based on cell metabolism; the non-metabolism dependent and metabolism-dependent. Depending on the location where the heavy metal ion is extracted from in the solution, biosorption could be arrayed as: (1) Extracellular accumulation (i) adsorption, (ii) ion exchange and complexation, (iii) Redox- reaction; (2) Intracellular accumulation absorption; and (3) Cell surface sorption/Precipitation: metabolic dependent transport.

Further, the past studies on biosorption detected that whenever there was the binding of metal ions on *Aspergillus niger, Fusarium oxysporum, Penicillium* sp., *Neurospora crassa*, the release of Ca^2+^ and Mg^2+^ was consequently triggered within the medium (Akthar et al., 1996).

## Factors Affecting Bioremoval of Copper and Cobalt Ions by Filamentous Fungi

### Effect of the Initial Concentration of Copper and Cobalt

Earlier studies have shown that the increase of biosorption capacity correlated with both fungal biomasses and the initial cobalt concentration. Cárdenas González et al. (2019) confirmed that the percentage of adsorption decreased whenever Co (II) concentration increased from 300 to 600 mg/L during the biosorption of cobalt ions by *Penicillium* spp. and *A. niger*. In similar biosorption studies, it has been reported that maximum Cu (II) and Co (II) uptake by *Aspergillus tamarii* NRC 3 biomass was 92.40 and 60%, respectively, in a solution comprising 5 g of *A. tamarii* wet biomass (Saad et al., 2019). It has also been observed that the removal capacity differs with fungal species. For example, during cobalt biosorption by filamentous fungi from the soil (*Trichoderma, Penicillium, Paecilomyces, Pythium, Rhizopus, Mortierella*, and *Aspergillus)*, the initial concentration of Co (II) resulted in increased removal capacity, achieving the maximum value of about 1036.8 μM of Co (II) uptake by *Mortierella* SPS 403 biomass at a maximum of 5.0 mM Co (II) concentration (Pal et al., 2006)

In previous studies on the tolerance of filamentous fungi to heavy metals, *Trichoderma atroviride* was found to tolerate high copper concentrations from 0 to 300 mg/L (López Errasquín and Vázquez, 2003). This correlates with the results by Romero et al. (2006), which suggested that the level of the metal accumulated is directly proportional to the heavy metal concentration due to the difference demonstrated by forces applied in the adsorption process. For example, with *Talaromyces helicus* (Raper and Fennell) C.r. Benj. (1955), it was remarked that the heavy metal uptake also increased from 100 to 200 mg/L with increasing Co (II) ion concentration from 100 to 600 ppm (Romero et al., 2006). This may be due to the high competition for the functional groups that appear on the surface of the fungal biomass ions (Cárdenas González et al., 2019).

Further studies by Tsekova and Todorova (2002) demonstrated that the microbial growth of *Aspergillus niger* B-77 related to the initial concentration of Cu (II). They have also shown that the metal uptake increased with the increase of Cu (II) concentrations. Where concentrations of 50, 100, and 200 mg/L Cu (II) ions were used for 24 h of cultivation, the accumulation levels were 3.7, 7.2, and 19.2 mg/g, respectively. A higher concentration of initial Cu (II) negatively affected the uptake of Cu (II). At 300 mg/L of Cu (II), neither microbial growth nor accumulation of Cu (II) was noticed. The maximum uptake was only observed at 200 mg/L of Cu (II) ions (Tsekova and Todorova, 2002).

### Effect of Initial Biomass Concentration for Copper and Cobalt Removal

**FIGURE 4:**
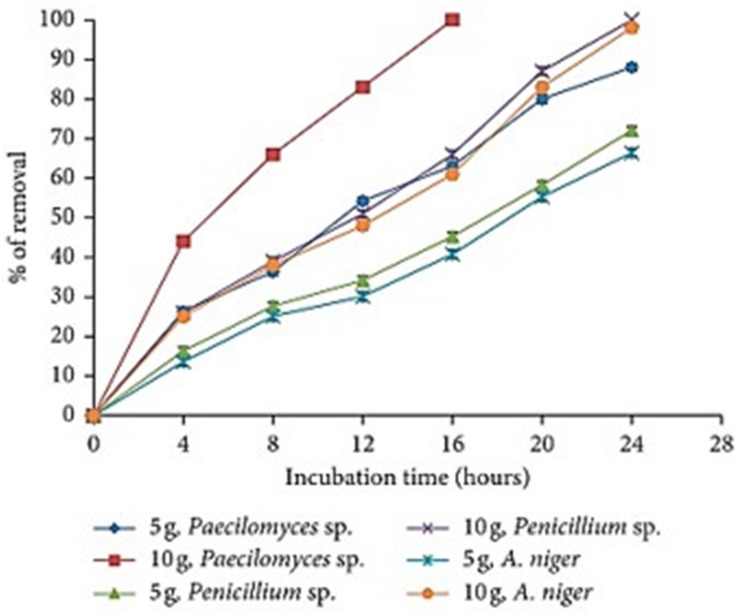
The effect of fungal biomass concentration on the removal of Co (II) (500 mg/L, 100 rpm, 28°C, pH 5.0, 24 h) Extracted from Cárdenas González et al. (2019).

Jayaraman and Arumugam (2014) demonstrated that the effect of the amount of biomass on the biosorption capacity is increased whenever the amount of fungal biomass is also increased. In the heavy metal solution, when 10 g rather than 5 g of the fungal biomass of *Paecilomyces* spp. was used, nearly 100% of the heavy metal was absorbed in only 16 h (Figure 4). Other filamentous fungi like *Penicillium* spp. and *Aspergillus niger* depicted high metal biosorption capacity at 24 h with 100 and 98% biosorption rates, respectively. In addition, Jayaraman and Arumugam (2014) demonstrated that *Aspergillus flavus* biomass correlated with the Cu (II) concentration. It was obvious that by increasing the amount of biomass from 1 to 10 mg/L, the metal removal capacity also extensively augmented with the highest recorded value of Cu (II) of 26.27 mg/g metal removal at 4 mg/mL of biosorbents. The increase in the removal capacity is mainly attributed to the amount of the added biosorbents fixed, the available number of binding sites and the adsorption surface area for metal biosorption (Figure 4) (Cárdenas González et al., 2019). Similar observations have been reported elsewhere with the same effect for *P. aeruginosa SPB-1* (Paraneeiswaran et al., 2014), and for *Cladonia rangiformis* hoffm (Horsfall and Spiff, 2005). The same was noted on the biosorption of Co (II) from aqueous solutions by *C. nucifera* L. (Hymavathi and Prabhakar, 2017).

The desorption percentage from Cu (II) accumulated biomasses was 80% under 0.1 N of NaOH and 0.1 N of HNO_3_, respectively. The regeneration of heavy metals and biosorbents by desorption is very crucial in applying the biosorption process industrially because it determines the cost of the entire process and the potential metal recovery performance (Ekmekyapar et al., 2006).

### Effect of pH and Incubation Time on the Removal of Copper and Cobalt

To understand the effect of both the incubation time and pH on biosorption of Co (II), 200 mg/L of the dried fungal biomass was used to determine the maximum biosorption in 24 h of incubation at pH 5.0 *Aspergillus niger, Penicillium* spp., and *Paecilomyces* spp. absorbed 93, 77.5, and 70.4% of Co (II), respectively (Cárdenas González et al., 2019). The above results concur with those obtained from an experiment with *Penicillium cyclopium* by Westling (1911) whereby the maximum biosorption of Cu and Co was also observed at pH 5 in 24 h of incubation time. It was also found that with *Paecilomyces catenlannulatus*, the uptake of Co augmented with the increasing pH from 4.5 to 7. The highest biosorption was recorded at pH 7.0. This was probably due to the Co (OH)_2_(s) precipitation (Li et al., 2014). This process can be justified by the fact that there was less competition among positively charged H^+^ and Co ^2+^ functional groups. So as the pH increases, additional ligands are displayed, and a higher number of negatively charged groups on the adsorbent matrix rise, improving the biosorption of every cationic species (Vafajoo et al., 2018).

Using *Aspergillus niger* for Cu (II) removal, multiple biomass samples were incubated at separate time intervals. The highest maximum removal capacity of *A. niger* was observed in 18 h of 25.2 mg/g Cu (II). Similar results have been reported with biosorption of Hg (II) by *Rhizopus oligosporus* Saito (1905) where the maximum biosorption was 33.33 mg/g at pH 6 under 6 h of incubation time (Ozsoy, 2010).

### The Effect of the Temperature on the Removal of Co (II) and Cu (II)

Temperature affects the biosorption of heavy metals. For the removal of Co (II), it has been shown that the highest adsorption capacity occurred at 50 ± 1°C: 100, 97.1, and 94.1%, for *Paecilomyces* sp., *Penicillium* sp., and *A. niger*, respectively, over 24 h. These reports are identical to those obtained for *P. catenlannulatus*, as the biosorption of Co (II) augmented with increasing temperature from 20 to 40°C (Cárdenas González et al., 2019).

During the removal of Cu (II), the biosorption capacity of *Aspergillus niger* was 26.2 mg/g at 31°C in the adsorption medium because the cell wall components stability is mostly influenced by temperature (Saad et al., 2019). It has also been reported that higher temperatures play a significant role in the enhancement of the adsorption ability of the fungal biomasses, which explains that in environments with elevated temperatures, such as in the tropics, it is favorable and practical to use fungal biomasses for bioremediation. High temperatures increase the activation of the adsorbing surfaces and enhance diffusivity of the heavy metal while catalyzing the movements of metal ions (Jayaraman and Arumugam, 2014; Cárdenas González et al., 2019).

## Summary and Prospects

Industrial wastewaters from mining and other industries released without treatments are harmful to the environment. Therefore, the environmental-friendly methods to dispose or recycle the wastewaters are among the emerging research areas, especially in the optimization of parameters (pH, incubation time, temperature, initial concentration, energy requirements, etc.) for metal ions removal and other contaminants from wastewaters. In developing countries, freshwater resources are scarce. Unfortunately, the available resources in mining areas are contaminated by high discharges of mining wastes into the aquatic systems posing a risk to both these ecosystems and human health (Drahansky et al., 2016; Mwaanga et al., 2019). The high Co (II) and Cu (II) concentrations, especially in mining and industrial wastes are among the substances of health concern since these metals are not biodegradable. Both tend to bioaccumulate in the living organisms and biomagnify along the food web resulting in several diseases and disorders (Drahansky et al., 2016).

The use of fungal biomasses for biosorption has attracted the attention of many researchers due to their numerous advantages (Table 3). According to data in Table 2, fungi have a high capacity to accumulate copper and cobalt. For advancements in bioremediation research, more fungal isolates should be obtained from copper and cobalt mining areas and investigated for their potential application. Their mechanisms of biosorption also warrants further research. In addition, the techniques to regenerate and use fungal biomass need further evaluation and improvement for deployment in areas which do not require advanced remediation technologies.

Due to the significant heavy metal pollution in developing countries that rely heavily on mining activities, a possible solution would be to use microorganisms for both biomining and bioremediation due to their eco-friendliness and cost-effectiveness. The information in this review shows that filamentous fungi have an exceptional capacity for exploration in the future for industrial-scale biosorption operations. Therefore, more understanding of microorganisms is needed, for instance in developing countries such as Zambia, one of the developing countries with nearly 6% of the total global reserve of copper and has among the world’s largest cobalt reserves that have been mined since the 1900s. There are minimum reports available of microbial and fungal diversity from the contaminated mining and other industrials sites (Staniland et al., 2010). Still, several reports have mentioned the accumulation of toxic heavy metals in the ecosystems (aquatic environments such as rivers, sediments), soil, atmosphere, and plants that can harmfully impact the human health, but also demonstrate the probability of high microbial diversity in such environments (Syakalima et al., 2001; Ikenaka et al., 2010; Elisha et al., 2012; Mugo and Town, 2013; Lindahl, 2014). Fortunately, according to studies conducted elsewhere in heavily contaminated sites, it was noticed that the long-term toxic impact of heavy metal pollutants usually increases the number of indigenous microbial diversities. In particular, bacteria and fungi in such areas were found with high tolerance potential to heavy metals due to the increased adaptation capacity over longtime exposure to contaminants. Some of the fungi are reported to thrive at high (toxic) metal ions concentrations (Bourceret et al., 2016). Therefore, future research should also focus on identifying and analyzing the potential of these microorganisms in similar sites for potential application in bioremediation.

Based on the data from this review, even though there is considerable knowledge about the fungal community in contaminated areas, there are still numerous gaps in developing countries with high reserves of minerals impacted by the severe effects of high contaminations due to traditional mining and handling of mining wastes. Therefore, further studies are necessary to fill the gaps in our scientific knowledge of these unidentified microbial communities. The wide research and development gap in advanced and environmentally friendly technologies for bioremediation of heavy metal contaminants should also be filled.

## Author Contributions

LD researched, wrote, and prepared the tables and figures for the first draft of this review. GK, CG, and KOO contributed to critical inputs, and designed and edited the last draft. All authors listed have made a substantial, direct and intellectual contribution to the work, and approved it for publication.

## Conflict of Interest

The authors declare that the research was conducted in the absence of any commercial or financial relationships that could be construed as a potential conflict of interest.
